# The associations between lumbar proprioception and postural control during and after calf vibration in people with and without chronic low back pain

**DOI:** 10.3389/fbioe.2024.1329437

**Published:** 2024-03-20

**Authors:** Zengming Hao, Xue Cheng, Haimei Jiang, Jiajia Yang, Yan Li, Wai Leung Ambrose Lo, Qiuhua Yu, Chuhuai Wang

**Affiliations:** ^1^ Department of Rehabilitation Medicine, The First Affiliated Hospital, Sun Yat-sen University, Guangzhou, China; ^2^ Department of Rehabilitation Medicine, The 10th Affiliated Hospital of Southern Medical University (Dongguan People’s Hospital), Dongguan, China; ^3^ Guangdong Engineering and Technology Research Center for Rehabilitation Medicine and Translation, the First Affiliated Hospital, Sun Yat-sen University, Guangzhou, China

**Keywords:** chronic low back pain, lumbar proprioception, postural control, passive joint repositioning sense, center of pressure, calf vibration

## Abstract

The relationships of lumbar proprioception with postural control have not been clarified in people with chronic low back pain. This study aimed to compare the associations between lumbar proprioception and postural control in response to calf vibration in individuals with and without chronic low back pain. In this study, we recruited twenty patients with chronic low back pain (CLBP group) and twenty healthy control subjects (HC group) aged between 18 and 50 years. This study was a cross-sectional study and completed from May 2022 to October 2022. The passive joint repositioning sense (PJRS) test for two positions (15° and 35°) were used to assess lumbar proprioception and expressed as the mean of reposition error (RE). Postural control was tested by adding and removing calf vibration while standing on a stable force plate with eyes closed. The sway velocity in the anterior-posterior (AP) direction of center of pressure (COP) data with a window of 15s epoch at baseline, during and after calf vibration was used to evaluate postural control. Mann-Whitney U-tests were used to compare the difference of lumbar proprioception between two groups, and the independent t-tests were used to compare the difference of postural control at baseline and during vibration, and a mixed design ANOVA was used to compare the difference of postural control during post-perturbation. In addition, to explore the association between postural control and lumbar proprioception and pain intensity, Spearman’s correlations were used for each group. The major results are: (1) significantly higher PJRS on RE of 15° (CLBP: 95% CI [2.03, 3.70]; HC: 95% CI [1.03, 1.93]) and PJRS on RE of 35° (CLBP: 95% CI [2.59, 4.88]; HC: 95% CI [1.07, 3.00]) were found in the CLBP group; (2) AP velocity was not different between the CLBP group and the HC group at baseline and during calf vibration. However, AP velocity was significantly larger in the CLBP group compared with the HC group at epoch 2–14 after calf vibration, and AP velocity for the CLBP group took a longer time (23 epochs) to return to the baseline after calf vibration compared with the HC group (9 epochs); (3) lumbar proprioception represented by PJRS on RE of 15°correlated negatively with AP velocity during and after vibration for the HC group. Within the CLBP group, no significant relationships between PJRS on RE for two positions (15° and 35°) and AP velocity in any postural phases were found. In conclusion, the CLBP group has poorer lumbar proprioception, slower proprioceptive reweighting and impaired postural control after calf vibration compared to the HC group. Lumbar proprioception offers different information on the control strategy of standing control for individuals with and without CLBP in the situations with proprioceptive disturbance. These results highlight the significance of assessing lumbar proprioception and postural control in CLBP patients.

## 1 Introduction

Chronic low back pain (CLBP) is the most common public health problem of all chronic pain conditions worldwide (Vlaeyen et al., 2018). CLBP not only seriously affects people’s quality of life and work, but also imposes a substantial economic burden on society (Foster et al., 2018; Hartvigsen et al., 2018). Patients with CLBP commonly exhibit abnormalities in the sensory, motor, and central nervous systems (Meier et al., 2019; Vlaeyen et al., 2018). Nonetheless, the source of pain remains unknown in most patients with CLBP (Fitzcharles et al., 2021; Maher et al., 2017). The effectiveness of different intervention methods for improving CLBP is not ideal, especially in the medium and long term. (Foster et al., 2018; Knotkova et al., 2021; Owen et al., 2020), which may be related to the unclear pathogenesis of CLBP and the mechanism of different intervention methods (Li et al., 2021). Recently, some suggest that muscle dysfunction may be the underlying cause of non-specific low back pain, which cannot be identified through traditional physical examination and history taking (Chiarotto and Koes, 2022; Knezevic et al., 2021). In fact, the low back pain-induced disrupted proprioceptive signaling plays an important role in the control of the motor system (Meier et al., 2019), and low back pain-induced reorganization of the motor cortex is associated with postural control deficit (Tsao et al., 2008). Therefore, accurately assessing the associations between lumbar proprioception and postural control is crucial for understanding the complex relationship between the altered sensory and motor systems in patients with CLBP.

It is well known that the proprioceptive information from different mechanoreceptors was integrated to control movement in daily movements (Han et al., 2016; Proske and Gandevia, 2012). Lumbar proprioception has been commonly investigated by passive joint repositioning sense (PJRS), active joint repositioning sense (AJRS), threshold to detect passive motion (TTDPM), and active movement extent discrimination assessment (AMEDA) in the people with CLBP (Lin et al., 2019). It is generally suggested that lumbar proprioception is impaired in the patients with CLBP compared with healthy subjects (Korakakis et al., 2021; Sakai et al., 2022), but the difference between groups is also affected by the proprioception testing methods (Lee et al., 2010). In fact, no differences in lumbar proprioception between the patients with CLBP and healthy control group were also found (Åsell et al., 2006; Lee et al., 2010). A literature review found that there is no significant relationship between pain and any proprioception measures. However, the subgroup analysis revealed that pain is significantly related to PJRS in trunk flexion with a small degree in a single study (Hu et al., 2017), while AJRS and TTDPM are not (Lin et al., 2019), suggesting that these measures of proprioceptive tests have varying levels of sensitivity. In addition, testing position has a significant effect on the acuity of lumbar spine position sense (Preuss et al., 2003). Thus, the precise assessment of lumbar proprioception require the consideration of multiple factors, such as testing position (sitting or standing), testing movement (flexion, extension, or side bending) and testing method (PJRS, AJRS, or TTDPM). Furthermore, a recent study found that people who developed low back pain after prolonged standing with 1 h exhibited relative higher proprioceptive weighting before the prolonged standing compared to those who did not develop pain (Orakifar et al., 2023) and suggested that proprioception deficit may be a cause for the development of low back pain. However, according to a recent systematic review, lumbar proprioception is impaired in patients with CLBP with only low certainty of evidence (Korakakis et al., 2021). Therefore, the relationship between lumbar proprioception and low back pain is very complicated, and the difference in lumbar proprioception between people with and without CLBP may also be influenced by other factors besides low back pain, which should be interpreted carefully.

Postural control is usually evaluated by biomechanical and neuromuscular parameters (Koch and Hänsel, 2019). Among them, the center of pressure (COP) data has been commonly used to assess postural control in people with and without CLBP (Ruhe et al., 2011a). The CLBP patients typically exhibit greater postural sway than controls, but less or similar postural sway is also found (Park et al., 2023), which may be related to pain intensity. For example, postural sway increases linearly with increasing pain intensity in CLBP patients with high pain intensity, while there is no significant difference in postural sway between healthy subjects and CLBP patients with low pain intensity (Ruhe et al., 2011b). The difference in postural control between people with and without CLBP in the static standing condition may be influenced by the challenge of testing conditions to some extent (Da et al., 2018). Additionally, calf vibration was found to shed insight into postural control affected by diseases or aging (Kiers et al., 2015; van den Hoorn et al., 2018). It has been demonstrated that the CLBP patients showed the diminished adaptive capability to vibration stimulus (Kiers et al., 2015), but the role of lumbar proprioception in postural control in patients with CLBP remains unknown. Therefore, the assessment of combination with lumbar proprioception and postural control in response to calf vibration would be beneficial for understanding the mechanism of CLBP and designing effective treatments.

This study aimed to investigate the associations between lumbar proprioception and postural control during and after calf vibration in people with and without CLBP. The hypotheses were: (1) lumbar proprioception deteriorates in the CLBP group; (2) postural stability declines in standing at baseline, during and after calf vibration in the CLBP group; (3) the relationships between lumbar proprioception and postural sway differs in people with and without CLBP.

## 2 Materials and methods

### 2.1 Participants

Twenty CLBP patients (CLBP group) and twenty healthy control subjects (HC group) aged between 18 and 50 years were recruited in this study. Because proprioception declines to some extent with aging, and the proprioceptive function of patients with chronic low back pain also declines (Sakai et al., 2022). In order to explore the decreased proprioception associated with chronic low back pain and its relationship with postural control, it is necessary to control for factor of age. We limit the sample to people under 50 years old of age referring to the relative higher effect of low back pain on global disability-adjusted life-years (DALYs) in the 25–49 years age group (Vos et al., 2020). The inclusion criteria for CLBP patients were medical diagnosis of non-specific low back pain and symptoms persisting for at least 3 months in the last year, with pain intensity score >2 according to the visual analog scale (VAS). The inclusion criteria for healthy control subjects were no history of low back pain in the last 12 months and during the time of visit. The exclusion criteria for all subjects were as follows: (1) spinal surgery or spinal fractures, (2) low back pain with neuropathy or radiculopathy, (3) neurological or musculoskeletal impairment. Differences of the demographic data between CLBP group and HC group were assessed using independent t-tests and Chi-squared test for gender. No significant differences were observed in age, gender, body weight, body height, body mass index (BMI) between the HC group and CLBP group. In addition, according to the classification of pain intensity in some previous studies (Asada et al., 2022; Sipko and Kuczyński, 2013), the average pain intensity of the CLBP group in this study was relatively lower (VAS<4). See details in Table 1. This study was approved by the Human Subjects Ethics Subcommittee of the first affiliated hospital of Sun Yat-sen University (issued no.2021886) and enrolled in the Chinese Clinical trial (registration number: ChiCTR2200064270). This study was conformed to the principles in Declaration of Helsinki. All subjects were recruited in this study were provided for written informed consent.

**TABLE 1:** Descriptive characteristics of the participants.

	HC group	CLBP group	*X* ^2^ or t	p
Age (years)	24.85 (3.48)	27.30 (5.03)	−1.791	0.081
Gender (male/female)	11/9	5/15	3.750	0.053
Body weight (kg)	59.85 (10.20)	58.03 (7.55)	0.643	0.524
Body height (cm)	167.43 (9.35)	163.80 (5.93)	1.465	0.151
Body mass index (kg/m^2^)	21.25 (2.44)	21.65 (2.81)	−0.488	0.628
Visual analog scale (0–10 cm)	0	3.88 (1.25)		

Notes: values are mean (standard deviations) or number. HC (healthy control). CLBP (chronic low back pain).

### 2.2 Sample size

The sample size was calculated using PASS software 15.0.5 based on the mean and standard deviation of center of pressure velocity in the anterior-posterior direction in the pilot stage of this study. To produce the power of 80% at an alpha level of 0.05, the sample size of 20 in each group was needed. This sample size has been shown to be adequate in previous postural control studies about chronic low back pain (Park et al., 2023).

### 2.3 Study design

The design of this study is a cross-sectional study. The study was completed from May 2022 to October 2022. All participants were recruited through advertisements. Prospective subjects were further consulted and diagnosed by a junior physician and a senior physician in the Rehabilitation Department of the first affiliated Hospital of Sun Yat-sen University. The same researcher is responsible for the lumbar proprioception test and postural control test of all subjects. Another researcher is responsible for all the later data analysis and statistics. No adverse reactions were reported in all subjects during the test.

### 2.4 Data collection

**FIGURE 1:**
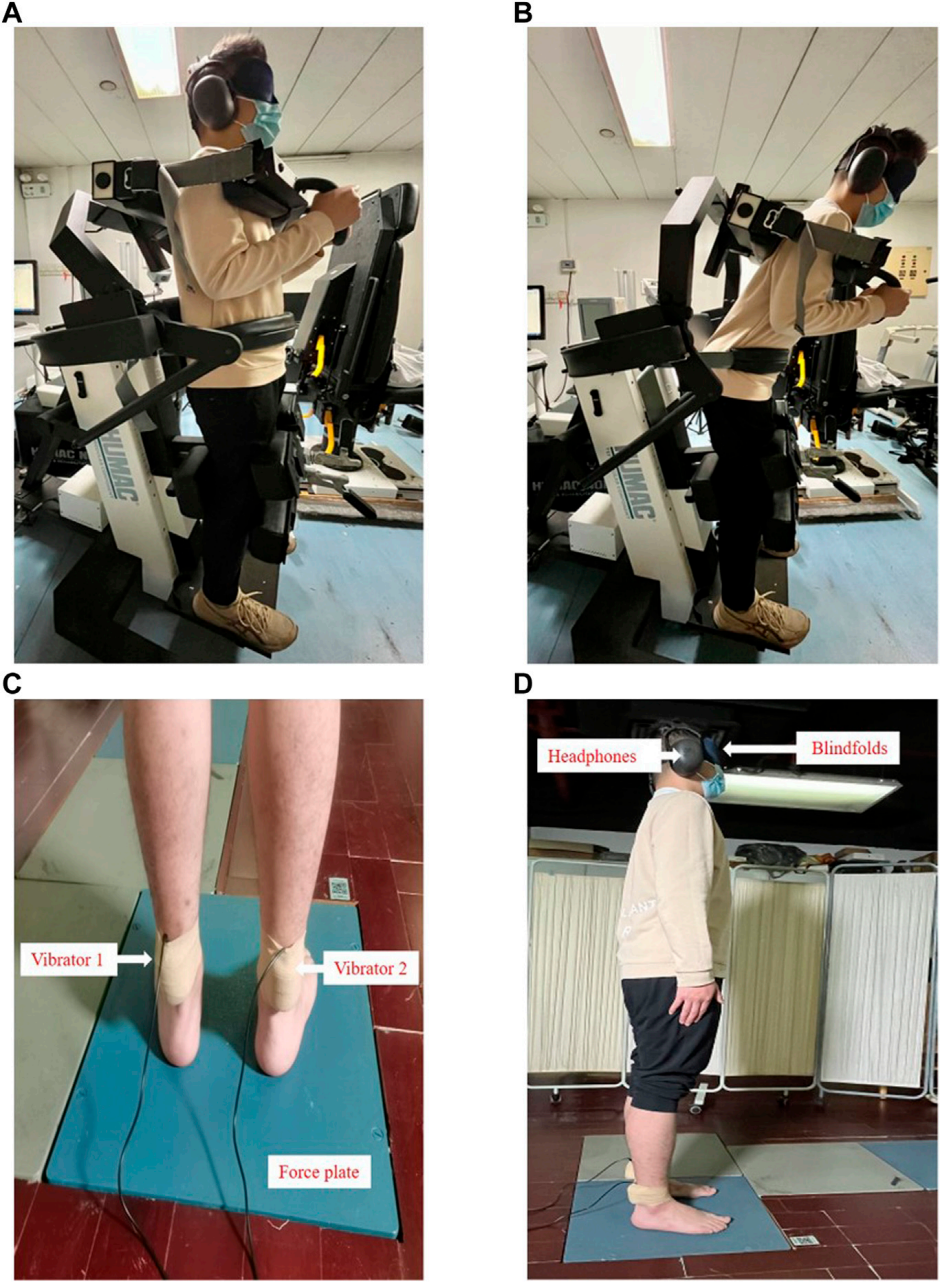
Lumbar proprioception test and postural control test: **(A)** Passive joint repositioning sense (PJRS) test in the neutral position; **(B)** Passive joint repositioning sense (PJRS) test in the target position; **(C)** Experimental setup of postural control test: standing on a force plate with vibrators on the triceps surae bilaterally; **(D)** Experimental setup of postural control test: standing on a force plate with wearing blindfolds and headphones.

**FIGURE 2:**
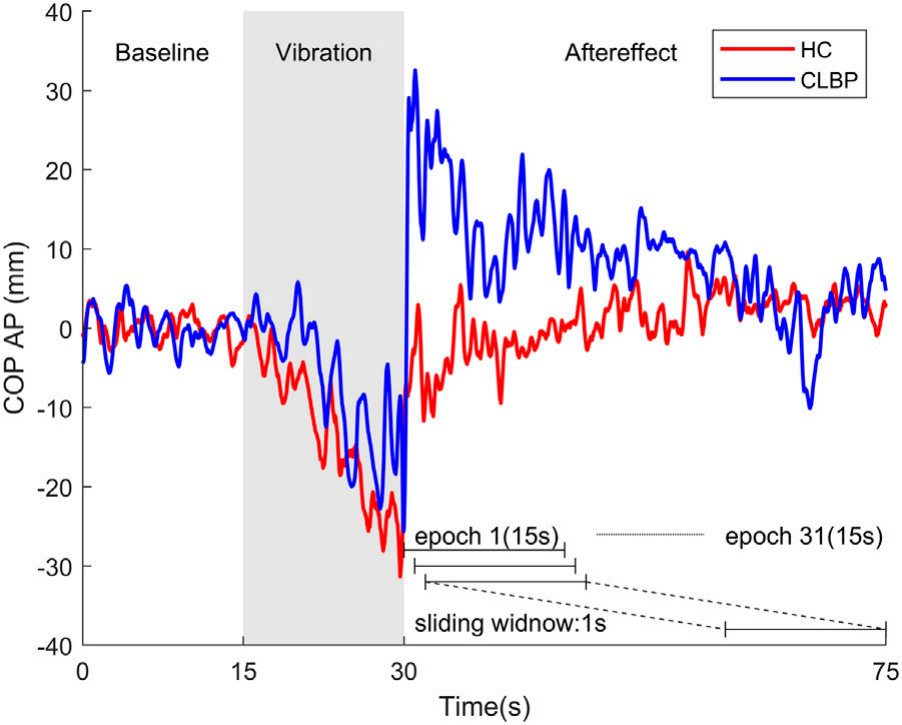
Examples of COP motion in the anterior-posterior (AP) direction from a representative healthy person (red line) and one patient with chronic low back pain (blue line) for three postural phases: baseline (15s), vibration (15s) and aftereffect (45s). AP velocity with 15-s epoch at baseline and during vibration were calculated, and AP velocity of 31 epochs with a sliding window of 1 s after vibration were also calculated.

All participants completed questionnaires of demographic characteristics and pain intensity of low back pain after providing written informed consent. The pain intensity was evaluated by the VAS, which consists of a 100 mm horizontal line from no pain (0) to worst imaginable pain (100). Then, they conducted the lumbar proprioception test and postural control test. Passive joint repositioning sense (PJRS) test was used to assess the lumbar proprioception with the Humac Norm system. Each participant was instructed to remember the target positions (15° and 35°) in trunk flexion, then moved from the neural position (Figure 1A) passively guided with a slow pace (1°/s). The participants reached the target position (Figure 1B) and then maintained the position for 5 s, and passively returned to the neural position subsequently. The PJRS test was repeated three times. During postural control test, all participants stood barefoot on a force plate (sampled at 1000Hz, AMTI, United States) with their arms to their sides, wearing blindfolds and headphones (Figure 1D) designed to reduce the potential effect of visual and auditory noise. Two custom-made muscle vibrators (frequency: 60Hz; amplitude: 1 mm) were attached on the triceps surae bilaterally (Figure 1C), the choice of vibration frequency and amplitude refer to a previous study (van den Hoorn et al., 2018). Data collection was started when the balance of participants had reached a steady state. Following the start of data collection, each participant was required to stand for 75 s. After the baseline of 15 s (BL), vibrators were switched on bilaterally for 15 s (VIB), and the following 45 s after vibration were recorded to assess the effects of post-vibration on standing balance (Figure 2).

### 2.5 Data analysis

For the PJRS test, Reposition error (RE) was measured in degrees and the average value of absolute error (AE) was taken as a measurement. For postural control test, The COP positions obtained by force plate were filtered using a 20 Hz low-pass, fourth-order, zero-lag Butterworth filter. Then, the COP data were decimated to 100 samples/s. As the mechanical vibration on calf mainly perturbs balance in the anterior-posterior (AP) direction (van den Hoorn et al., 2018), so further analysis were focused on COP motion in the AP direction. Owing to the mean velocity was found as the most sensitive parameter for discriminating people with and without CLBP (Mohammadi et al., 2021), and the analysis using a sliding window with 1 s after removal of vibration has been found to reduce the variance than the analysis using one epoch during or after vibration (van den Hoorn et al., 2018). Thus, AP velocity was used for assessment of postural control and applied in a windowed (15-s epoch) manner at three postural phases (baseline, vibration and aftereffect) in this study. Therefore, AP velocity at baseline and during vibration were calculated, and AP velocity of 31 epochs with a sliding window of 1s after vibration were also calculated to assess the dynamic changes of aftereffect of vibration (Figure 2).

### 2.6 Statistics

All statistical testing was conducted using SPSS (IBM SPSS Statistics, Version 25, SPSS Inc., Chicago, IL, United States). Each measure’s distribution of normality was tested (Shapiro-Wilk test, *p* > 0.05). Mann-Whitney U-tests were utilized for PJRS on RE of 15° and 35° to explore the differences of lumbar proprioception between two groups. Differences of AP velocity at baseline and during vibration between groups were assessed using independent t-tests respectively. For AP velocity after vibration, a linear mixed design ANOVA with between-subject factor (group) and within-subject factor (time window) was conducted to assess the main effect of group and time window, and the significant interaction was explored using simple effects analysis. A Greenhouse-Geisser correction was applied when the assumption of sphericity could not be upheld (Mauchly’s test, *p* < 0.05). The duration of the aftereffect for each group was assessed using paired-sample t-tests comparing each 15-s window epoch after vibration with the baseline. To explore the association between lumbar proprioception and postural control in the HC group and CLBP group, Spearman’s correlations were computed to determine the relationship between the PJRS on RE for two positions (15° and 35°) and AP velocity at baseline, during and after vibration for each group respectively. In addition, to explore the association between pain intensity and lumbar proprioception and postural control in the CLBP group, Spearman’s correlations were also computed to determine the relationship between the VAS and PJRS on RE for two positions (15° and 35°) and AP velocity at baseline, during and after vibration separately. In the stage of post-vibration, the mean AP velocity of the average time window (epoch) required for each group to return to the baseline level was selected. The significance level was set as *p* < 0.05 with two-tailed. All *p* values were corrected using Bonferroni method. Effect size values (η_p_^2^) were reported for ANOVA.

## 3 Results

### 3.1 Lumbar proprioception

Table 2 provides the results of lumbar proprioception between the HC group and CLBP group. Compared with the HC group, the CLBP group showed significantly higher PJRS on RE of 15° and PJRS on RE of 35°.

**TABLE 2:** Lumbar proprioception between HC group and CLBP group.

	HC group	CLBP group	*z*	p
PJRS on RE of 15°(degrees)	1.48 (1.03, 1.93)	2.87 (2.03, 3.70)	−2.790	0.005
PJRS on RE of 35°(degrees)	2.18 (1.37, 3.00)	3.73 (2.59, 4.88)	−2.470	0.014

Notes: values are mean (95% CI, lower limit, 95% CI, upper limit), HC (healthy control). CLBP (chronic low back pain).

### 3.2 Postural control at baseline, during and after vibration

**FIGURE 3:**
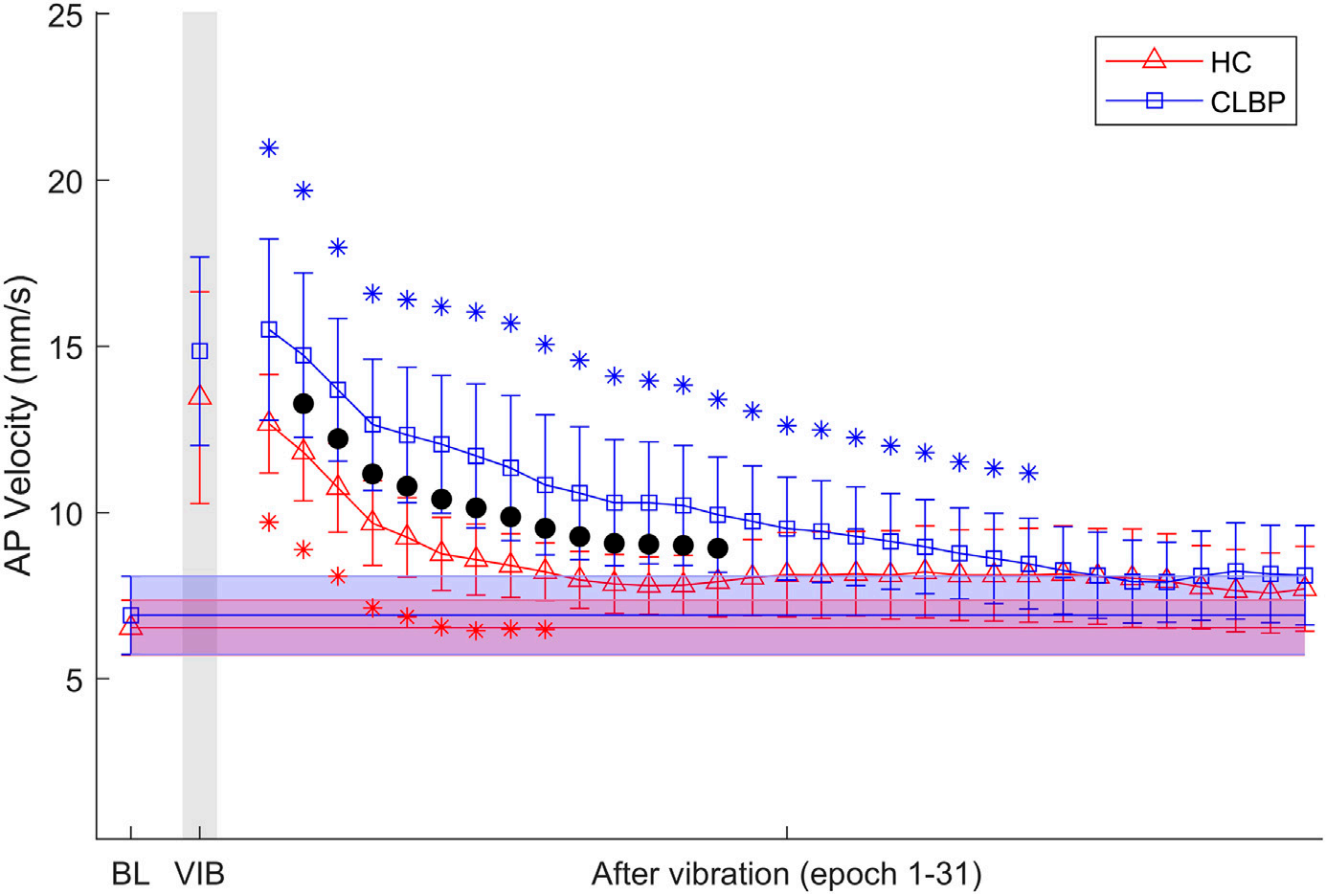
Results of mean AP velocity at baseline (BL), during vibration (VIB) and after vibration epochs (epoch 1–31) for the HC group and CLBP group. Note that black dots denote the significant group difference showed by simple effects analysis after ANOVA with time window as within-subject factor and group as between-subject factor. Shaded bars represent the mean and 95% confidence interval of AP velocity at baseline for the HC group (red) and CLBP group (blue). * Significant difference from baseline mean for the HC group (red) and CLBP group (blue), showed by paired t-tests with alpha level corrected for multiple comparisons (*p* < 0.05/31). Error bars represent ±95% confidence interval.

Figure 3 illustrates the mean AP velocity during three postural phases in the HC group and CLBP group. No significant differences were observed between groups at baseline (*p* = 0.589) and during vibration (*p* = 0.498). A mixed repeated ANOVA showed that AP velocity declined over time as showed by a main effect of time window within the phase of post-vibration (F (2.435,92.520) = 31.546, *p* < 0.001, η_p_^2^ = 0.454), and AP velocity in the both groups changed in different ways over time as showed by a significant time window × group interaction (F (2.435,92.520) = 4.347, *p* = 0.011, η_p_^2^ = 0.103). Simple effects analysis demonstrated that there was significantly larger AP velocity for the CLBP group compared with the HC group at epoch 2–14 (*p* = 0.006–0.044). No overall group difference was found within post-vibration (*p* = 0.074). In addition, the durations of aftereffects were examined with paired-sample t-tests comparing each 15-s window epoch with the baseline, with an alpha level corrected for multiple comparisons to 0.05/31. Tests showed that for the HC group the aftereffect was significant from baseline in the first 9 epochs (t (19) = 4.528–8.792, *p* < 0.001), while the aftereffect was significant from baseline in the first 23 epochs in the CLBP group (t (19) = 3.729–8.222, *p* ≤ 0.001).

### 3.3 Relationships between postural control and lumbar proprioception and pain intensity

**FIGURE 4:**
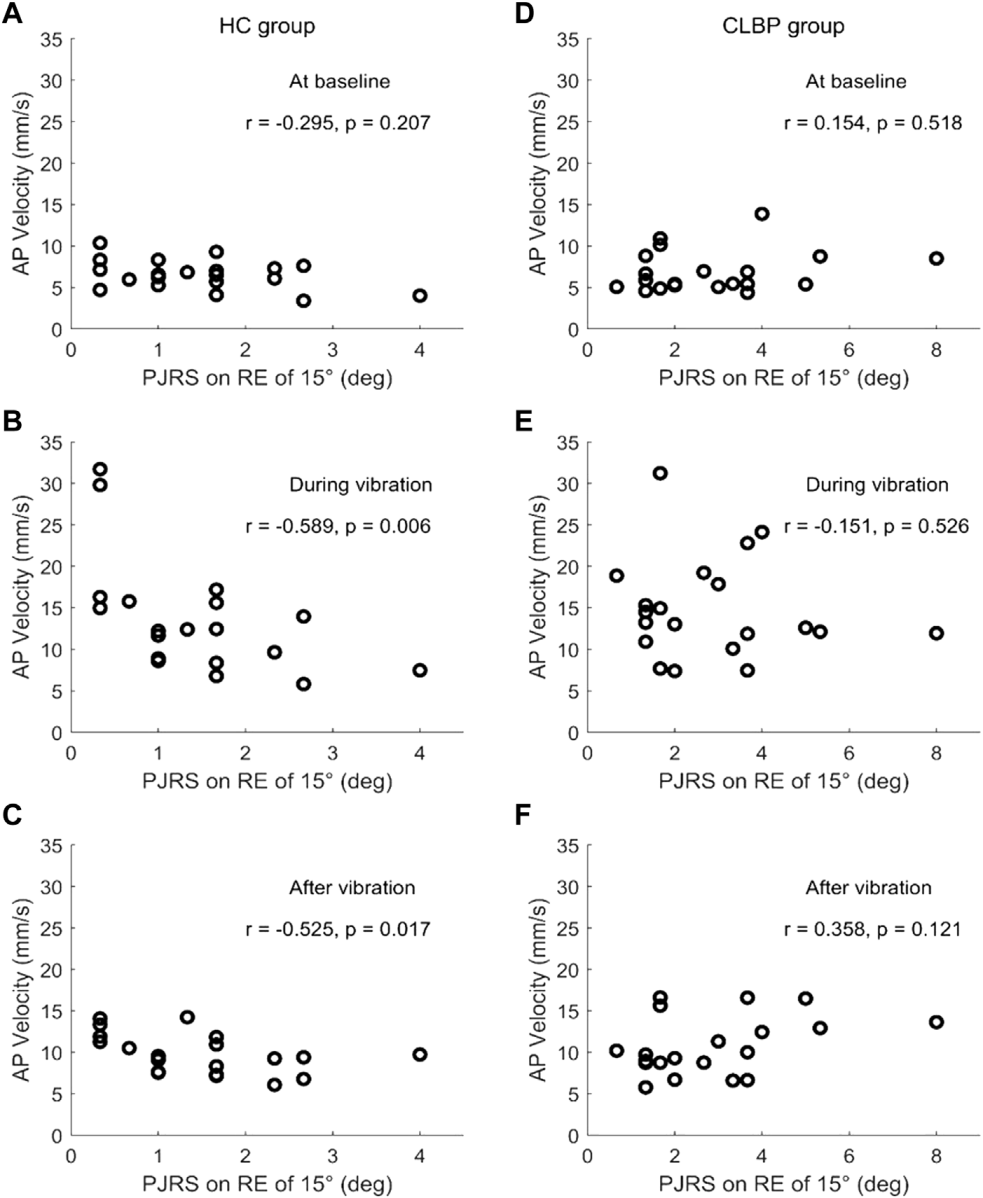
Scatter plots of the relation of PJRS on RE of 15° and AP velocity at baseline **(A, D)**, during vibration **(B, E)** and after vibration **(C, F)** for the HC group (left) and CLBP group (right).

Table 3 shows the results of relationships between postural control and lumbar proprioception in the CLBP group and HC group. In the HC group, a significant negative relationship between PJRS on RE of 15° and AP velocity during vibration was observed (*p* = 0.006, 95% CI [-0.791, −0.270]). In contrast, no statistically significant relationships between PJRS on RE of 15° and AP velocity at three postural phases in the CLBP group were found (*p* > 0.05). The plots in Figure 4 show that the smaller the PJRS on RE of 15°, the larger the AP velocity during vibration in the HC group (Figure 4B), and this relationship also existed after vibration (*p* = 0.017, 95% CI [-0.754, −0.182]) (Figure 4C). However, no such discernible trend was observed at any postural phases in the CLBP group (Figures 4D–F). No significant relationships were also found between PJRS on RE of 35° and AP velocity at baseline, during and after vibration (*p* > 0.05) for any group. In addition, according to the results of relationships between pain intensity and lumbar proprioception and postural control in the CLBP group, no significant relationships were found between VAS and PJRS on RE of 15°, PJRS on RE of 35° and AP velocity at three postural phases (baseline, during vibration and after vibration) for the CLBP group (Table 4).

**TABLE 3:** The correlations of postural control and lumbar proprioception in the HC group and CLBP group.

	HC				CLBP			
Variables	PJRS^1^		PJRS^2^		PJRS^1^		PJRS^2^	
	r	p	r	p	r	p	r	p
Vel_BL	−0.295	0.207	0.139	0.559	0.154	0.518	0.436	0.055
Vel_VIB^1^	−0.589	**0.006**	−0.087	0.716	−0.151	0.526	−0.080	0.737
Vel_VIB^2^	−0.525	0.017	0.216	0.360	0.358	0.121	0.302	0.195

VAS: visual analog scale; HC: healthy control; CLBP: chronic low back pain. PJRS^1^ and PJRS^2^ denote PJRS, on RE, of 15° and PJRS, on RE, of 35°. Vel_BL, Vel_VIB^1^ and Vel_VIB^2^ denote AP, velocity at baseline, during vibration and after vibration respectively; r: Spearman’s correlation coefficient. Bold *p* values represent p < adjusted significance level (0.05/3), shaded cells denote significant Spearman’s correlation coefficient.

**TABLE 4:** The correlations of pain intensity and lumbar proprioception and postural control in the CLBP group.

	VAS-PJRS^1^	VAS-PJRS^2^	VAS-Vel_BL	VAS-Vel_VIB^1^	VAS-Vel_VIB^2^
r	0.082	0.067	0.203	0.220	0.146
p	0.730	0.780	0.391	0.352	0.540

VAS: Visual analog scale. PJRS^1^ and PJRS^2^ denote PJRS, on RE, of 15° and PJRS, on RE, of 35°. Vel_BL, Vel_VIB^1^ and Vel_VIB^2^ denote AP, velocity at baseline, during vibration and after vibration respectively; r: Spearman’s correlation coefficient.

## 4 Discussion

The main findings are as follows: (1) significantly higher PJRS on RE of 15° and PJRS on RE of 35° were found in the CLBP group compared with the HC group; (2) AP velocity was not different between the CLBP group and HC group at baseline and during calf vibration. However, AP velocity was significantly larger in the CLBP group compared with the HC group at epoch 2–14 after calf vibration, and AP velocity for the CLBP group took a longer time (23 epochs) to return to the baseline compared with the HC group (9 epochs); (3) lumbar proprioception represented by PJRS on RE of 15° correlated negatively with AP velocity during vibration for the HC group, while this relationship did not existed within the CLBP group. No significant relationships were found between VAS and PJRS on RE of 15°, PJRS on RE of 35° and AP velocity at three postural phases in the CLBP group. These results are discussed below.

### 4.1 Diminished lumbar proprioception in the CLBP group

Significantly higher PJRS on RE of 15° and 35° were found in the CLBP group, demonstrating diminished lumbar proprioception in the CLBP group compared with the HC group, which was consistent with our previous study (Cheng et al., 2023). In fact, many studies have found the poor lumbar proprioception in the CLBP group (Brumagne et al., 2000; Sakai et al., 2022; Sheeran et al., 2012), which may be due to the altered paraspinal muscle spindle afference and central processing (Brumagne et al., 2000). On the other hand, no differences in the repositioning tests between CLBP patients and healthy subjects was also found (Åsell et al., 2006). In addition, significantly greater motion perception threshold was found in CLBP group compared with controls, but no significant difference between groups in the repositioning tasks (Lee et al., 2010), which may be contributed to the sensitivities of different indicators of proprioception tests (Han et al., 2016). Furthermore, decreased ankle joint proprioception (Xiao et al., 2022) and decreased knee joint proprioception (Ranjbar et al., 2023) were also found in CLBP group compared with controls, which indicated that the diminished lumbar proprioception in patients with CLBP is more likely related to abnormal central processing. Furthermore, a previous study has found that people who developed low back pain after prolonged standing exhibited altered proprioceptive postural control before the prolonged standing compared to those who did not develop pain and suggested that proprioception deficit may be causal for the development of low back pain (Orakifar et al., 2023). Moreover, the symptoms experienced in healthy people during the prolonged standing are like symptoms typically experienced by people with low back pain (Sorensen et al., 2015). These results showed that the proprioception should be considered for the assessment of the patients with chronic low back pain. Thus, lumbar proprioception is impaired in patients with CLBP, the differences in lumbar proprioception may not be clinically meaningful (Korakakis et al., 2021), so accurate assessment of proprioception at multiple joints in lower limbs and trunk is necessary and important for establishing a precise treatment procedure (Sakai et al., 2022).

### 4.2 Postural control between the CLBP and HC group at baseline

A new meta-analysis suggests that CLBP is associated with increased postural sway, especially in the situation when vision was occluded (Park et al., 2023). However, the present result indicated that CLBP caused no significant difference of AP velocity at baseline. In fact, no significant differences between the CLBP group and HC group in the static stance were found in many studies (Brumagne et al., 2008; Della Volpe et al., 2006; Koch et al., 2020), which may be contributed the low level of pain intensity in the CLBP group (Ruhe et al., 2011b) or the sensitivity of different COP parameters for assessing postural control (Kiers et al., 2015) or the challenge of testing conditions (Da et al., 2018). For example, postural sway increases linearly with increasing perceived pain intensity greater than 4 on an NRS scale, while postural sway between healthy subjects and CLBP patients with lower pain intensity (NRS = 2) show no differences (Ruhe et al., 2011b). Moreover, some studies found less postural sway in CLBP patients with lower pain intensity compared with healthy subjects (Lafond et al., 2009; Mok et al., 2004; Salavati et al., 2009). In addition, subjects with CLBP showed similar postural sway as subjects without CLBP, but frequency and irregular measures (such as entropy and fractals) differ between groups (Kiers et al., 2015). Patients with CLBP commonly exhibited greater postural sway under the challenging conditions (Da et al., 2018; Della Volpe et al., 2006). Therefore, postural sway increases in some but not all patients with CLBP (Mazaheri et al., 2013), which may be correlated with many factors.

### 4.3 Effect of vibration on postural control

The time window × group interaction indicates that AP velocity in both groups vary over time window, and AP velocity was significantly larger in the CLBP group compared with the HC group at epoch 2–14 after calf vibration, which showed that postural control is impaired after calf vibration in CLBP patients compared with healthy subjects and using sliding window after vibration have reduced the variance leading to significant differences between groups (van den Hoorn et al., 2018). The CLBP group took a longer time to return to the baseline compared with the HC group, which confirmed that the CLBP patients exhibit slower balance recovery after perturbation, consistent with previous studies (Kiers et al., 2015; Mok et al., 2011). However, there was no difference of AP velocity during calf vibration between groups, which may be contributed to proprioceptive weighting change in patients with CLBP (Brumagne et al., 2004), and the asymmetry of reweighting dynamics with slower sensory reweighting following a high-to-low transition compared with a low-to-high transition (Assländer and Peterka, 2014). This suggests that when vibration is applied from the baseline, it affects postural control less compared to when vibration is ceased. Postural sway may be used by central nervous system as an exploratory role to ensure continuous dynamic inputs from multiple sensory systems (Carpenter et al., 2010; Murnaghan et al., 2011). Thus, these results shown that the CLBP patients and healthy subjects may use different postural control strategy during and after calf vibration (Brumagne et al., 2008).

### 4.4 Relationships between postural control and lumbar proprioception differs in the CLBP and HC group

According to the results of relationships between PJRS on RE of 15° and AP velocity at baseline, during and after calf vibration for the HC group and CLBP group, it was found that lumbar proprioception represented by PJRS on RE of 15°correlated negatively with AP velocity during vibration and after vibration (not corrected using Bonferroni method) in the HC group, which could be explained by the exploratory role of postural sway (Carpenter et al., 2010; Murnaghan et al., 2011). In contrast, no significant relationships between lumbar proprioception and postural control existed in the CLBP group, which consistent with the previous study (Reeves et al., 2009). In fact, a previous study found positive relationship between overall stability index for the left foot and absolute error score of shoulder proprioception in healthy controls, while no such relationship existed in patients with chronic ankle instability (Springer et al., 2015), another study found that proprioception were correlated with Berg Balance Scale among older adults aged 65–74 years, but non among the elderly aged over 75 years (Wang et al., 2022), which further confirmed that patients with CLBP have altered postural control strategies during standing balance compared with healthy subjects. However, no significant relationships were found between PJRS on RE of 35° and AP velocity at baseline, during and after vibration for both groups, which may be related with the sensitivity of different indicators of proprioception tests. In addition, there was no significant association between lumbar proprioception and postural control at baseline for both groups, which demonstrated that the relationship varies and depend on the specific sensory conditions. A review has concluded that poor proprioception was one of the main causes of decreased postural control in elderly patients with low back pain (Sakai et al., 2022). Recent studies also found that fear of movement (Meinke et al., 2022) and functional disability (Sun et al., 2023) were significantly correlated with postural sway in patients with CLBP. Thus, the negative relationship between postural sway and lumbar proprioception in healthy subjects may be explained as an exploratory role of postural sway for acquiring effective proprioceptive information to maintain standing balance, while the ability of postural sway as an exploratory role may be impaired in the CLBP group.

### 4.5 Relationships between pain intensity and lumbar proprioception and postural control in the CLBP group

According to the results of relationships between pain intensity and lumbar proprioception in the CLBP group, no significant associations were found, which was consistent with the results of two recent systematic reviews (Ghamkhar and Kahlaee, 2019; Lin et al., 2019). However, the measurement of lumbar proprioception using PJRS showed a small correlation with pain intensity (Lin et al., 2019). Thus, the absence of a moderate to strong associations between lumbar proprioception and pain intensity challenge the idea that lumbar proprioception deficit is a cause or consequence of chronic low back pain (Lin et al., 2019). This phenomenon also means that there may be other factors causing lumbar proprioception deficits and chronic low back pain, which require further study. In addition, no significant relationships between pain intensity and postural control at different postural phases were found in the CLBP group, which was similar to a previous study (Sipko and Kuczyński, 2013). Moreover, no significant relationships between pain intensity and postural control were observed in low pain (NRS = 0–3) CLBP group or high pain (NRS = 4–10) CLBP group (Sipko and Kuczyński, 2013). However, another study found that COP velocity increases linearly with increasing pain intensity when pain intensity were greater than 4 on a NRS scale (Ruhe et al., 2011b). The CLBP patients recruited in this study had relatively low level of mean pain intensity (3.88 on a VAS scale), which partly explains the lack of a significant correlation between pain intensity and postural control in the CLBP group. Therefore, the relationships between pain intensity and lumbar proprioception and postural control in the CLBP group seem to be more complex.

### 4.6 Limitations

Lumbar proprioception was only tested in this study. However, human mainly used the ankle strategy in the non-challenging standing tasks, and some studies found that knee proprioception (Ranjbar et al., 2023) and ankle proprioception (Xiao et al., 2022) changed significantly in the CLBP group compared with HC group. These studies confirmed that proprioceptive input from multiple joints contributed to the control of standing balance, especially for the contribution of ankle proprioception to postural control (Fu and Hui-Chan, 2005). Thus, it cannot be confirmed whether the same relationships exists between ankle and knee joint proprioception and postural control in this study. In addition, significantly differences of postural control were observed among different subgroup with different pain intensity of CLBP patients in many studies (Ruhe et al., 2011b), so the lack of a significant difference in postural control between groups at baseline and during vibration may be partly affected by the lower level of pain intensity in the patients in this study.

### 4.7 Future recommendations

Based on the distinct associations between lumbar proprioception and postural control during calf vibration in people with and without CLBP which were found in this study, and significant differences of knee proprioception and ankle proprioception existed between the CLBP group and HC group, thus assessing the proprioception across multiple joints (knee, ankle and lumbar proprioception) and the associations between different proprioception and postural control in CLBP patients are recommended in the future work. In addition, the myoelectric manifestations of different associations between postural control and lumbar proprioception in both groups also need further exploration. Furthermore, the effect of pain intensity on the associations between proprioception and postural control in patients with CLBP should also be considered in the further research.

## 5 Conclusion

The patients with CLBP have poorer lumbar proprioception, slower proprioceptive reweighting and impaired postural control after calf vibration compared with healthy subjects. In addition, lumbar proprioception provides different information on the control strategy of standing balance between people with and without CLBP in the situation with proprioceptive disturbance.

## Data Availability

The raw data supporting the conclusion of this article will be made available by the authors, without undue reservation.
